# Guanidine Alkaloids from the Marine Sponge *Monanchora pulchra* Show Cytotoxic Properties and Prevent EGF-Induced Neoplastic Transformation in Vitro

**DOI:** 10.3390/md14070133

**Published:** 2016-07-15

**Authors:** Sergey A. Dyshlovoy, Kseniya M. Tabakmakher, Jessica Hauschild, Regina K. Shchekaleva, Katharina Otte, Alla G. Guzii, Tatyana N. Makarieva, Ekaterina K. Kudryashova, Sergey N. Fedorov, Larisa K. Shubina, Carsten Bokemeyer, Friedemann Honecker, Valentin A. Stonik, Gunhild von Amsberg

**Affiliations:** 1 Laboratory of Experimental Oncology, Department of Oncology, Hematology and Bone Marrow Transplantation with Section Pneumology, Hubertus Wald-Tumorzentrum, University Medical Center Hamburg-Eppendorf, Martinistrasse 52, Hamburg 20246, Germany; j.hauschild@uke.de (J.H.); k.otte@uke.de (K.O.); c.bokemeyer@uke.de (C.B.); friedemann.honecker@zetup.ch (F.H.); g.von-amsberg@uke.de (G.v.A.); 2 Laboratory of Marine Natural Products Chemistry, G.B. Elyakov Pacific Institute of Bioorganic Chemistry, Far-East Branch, Russian Academy of Sciences, Prospect 100-let Vladivostoku 159, Vladivostok 690022, Russia; xen-tab@yandex.ru (K.M.T.); flagmandv@inbox.ru (R.K.S.); gagry@rambler.ru (A.G.G.); makarieva@piboc.dvo.ru (T.N.M.); catrinog.81@mail.ru (E.K.K.); fedorov@piboc.dvo.ru (S.N.F.); shubina@piboc.dvo.ru (L.K.S.); stonik@piboc.dvo.ru (V.A.S.); 3 School of Natural Sciences, Far Eastern Federal University, Sukhanova Street 8, Vladivostok 690091, Russia; 4 Tumor and Breast Center ZeTuP St. Gallen, Rorschacher Strasse 150, St. Gallen 9006, Switzerland

**Keywords:** guanidine alkaloids, monanchocidin A, *Monanchora pulchra*, apoptosis, MAPK, AP-1, neoplastic transformation

## Abstract

Guanidine alkaloids from sponges *Monanchora* spp. represent diverse bioactive compounds, however, the mechanisms underlying bioactivity are very poorly understood. Here, we report results of studies on cytotoxic action, the ability to inhibit EGF-induced neoplastic transformation, and the effects on MAPK/AP-1 signaling of eight rare guanidine alkaloids, recently isolated from the marine sponge *Monanchora pulchra*, namely: monanchocidin A (**1**), monanchocidin B (**2**), monanchomycalin C (**3**), ptilomycalin A (**4**), monanchomycalin B (**5**), normonanchocidin D (**6**), urupocidin A (**7**), and pulchranin A (**8**). All of the compounds induced cell cycle arrest (apart from **8**) and programmed death of cancer cells. Ptilomycalin A-like compounds **1**–**6** activated JNK1/2 and ERK1/2, following AP-1 activation and caused p53-independent programmed cell death. Compound **7** induced p53-independent cell death without activation of AP-1 or caspase-3/7, and the observed JNK1/2 activation did not contribute to the cytotoxic effect of the compound. Alkaloid **8** induced JNK1/2 (but not ERK1/2) activation leading to p53-independent cell death and strong suppression of AP-1 activity. Alkaloids **1**–**4**, **7**, and **8** were able to inhibit the EGF-induced neoplastic transformation of JB6 P^+^ Cl41 cells. Our results suggest that investigated guanidine marine alkaloids hold potential to eliminate human cancer cells and prevent cancer cell formation and spreading.

## 1. Introduction

Guanidine alkaloids from sponges belonging to the genus *Monanchora* represent compounds with diverse chemical structures and a broad spectrum of biological activities [1,2,3,4,5,6]. Some of these compounds are cytotoxic against different types of human cancer cells [7,8,9,10,11,12,13]. Recently, we have reported on one of these compounds, monanchocidin A, to possess a unique and novel combination of properties. Remarkably, we have identified an unusual mode of action mediating the observed cytotoxic activity [8,10,14] of this natural compound: thus, monanchocidin A was able to induce cytotoxic autophagy (type II programmed cell death) and lysosomal membrane permeabilization (LMP) in human germ cell tumor cells [14]. In addition, it exhibited anti-migratory activity at low non-cytotoxic concentrations [15]. At the same time, the classical apoptosis, which is frequently activated by conventional chemotherapies and which has been initially suggested as a main mechanism of monanchocidin A cytotoxicity [8], was of minor importance in this case [14].

In addition to monanchocidin A, very little is known on bioactivity, and literally nothing is known on the mechanisms of action of other guanidine alkaloids from marine sponges *Monanchora* spp. Ptilomycalin A (**4**) has been initially isolated by Kashman et al. in 1989 from the Caribbean sponges *Ptilocausis spiculifer* and from a Red Sea sponge *Hemimycale* sp. [16]. Recently, we were able to isolate it from the marine sponge *Monanchora pulchra* [17]. This alkaloid has shown a potent cytotoxicity against a broad panel of human cancer cells [16,18,19,20]. However, to date molecular targets and the mode of action of ptilomycalin A in mammalian cell have not been studied. For compounds **2**, **3**, and **5**–**8**, isolation and preliminary results on cytotoxicities have been recently reported by us [9,10,11,17,21,22]. However, no data on their mode of cytotoxic action are available so far.

Crambescidin alkaloids are another group of natural guanidine alkaloids structurally related to ptilomycalin A. Crambescidins are cytotoxic against different human cancer cells, as well as yeast cells, and induce cell cycle arrest [23,24,25]. They induce differentiation of K562 chronic myelogenous leukemia cells [23] and block Ca^2+^, Na^+^, and K^+^ channels [26,27]. Recently, Rubiolo et al. reported the results of transcriptomic analysis of HepG2 human cancer cells treated with crambescidin-816 at non-cytotoxic concentrations [28]. They were able to demonstrate a decreased cancer cell migration by inhibition of cell-cell and cell-matrix adhesion, a reduced tight junctions formation, and the alteration of cytoskeleton dynamics [28].

**Figure 1:**
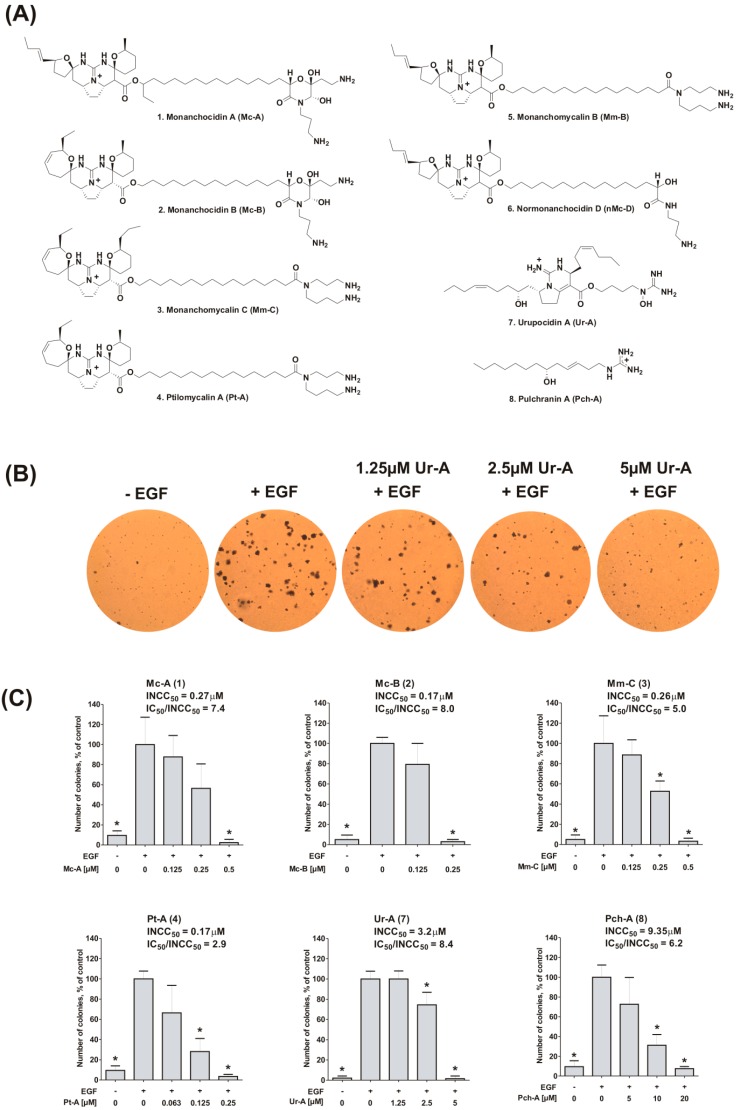
Alkaloids **1**–**8** and their effect on EGF-induced neoplastic transformation of JB6 P^+^ Cl41 cells. (**A**) Structures of guanidine alkaloids **1**–**8** isolated from the marine sponge *Monanchora pulchra*; (**B**) representative pictures of microscopic fields of EGF-induced colonies of JB6 P^+^ Cl41 cells in soft agar treated with urupocidin A (**6**) at the indicated concentrations; (**C**) Inhibition of EGF-induced neoplastic transformation of JB6 P^+^ Cl41 cells by compounds **1**–**4**, **7**, and **8**. INCC_50_—concentration leading to a 50% inhibition of colonies formation. Ratios of IC_50_/INCC_50_ were calculated using the IC_50_ values from Table 1 generated by using the MTT assay. * indicates a statistically significant difference (*p* < 0.05) from the control value.

In continuation of our study of marine compounds possessing potential cancer-preventive, cytotoxic and anti-cancer properties [29,30,31,32,33,34,35,36,37,38], we investigated the in vitro activity and mode of action of eight rare marine guanidine alkaloids, which have recently been isolated in our laboratory from the marine sponge *Monanchora pulchra*, namely: monanchocidin A (Mc-A, **1**) [8], monanchocidin B (Mc-B, **2**) [10], monanchomycalin C (Mm-C, **3**) [17], ptilomycalin A (Pt-A, **4**) [16], monanchomycalin B (Mm-B, **5**) [9], and normonanchocidin D (nMc-D, **6**) [22], urupocidin A (Ur-A, **7**) [21], and pulchranin A (Pch-A, **8**) [11] (Figure 1A). Here, we report the in vitro cancer chemo-preventive properties, the cytotoxic activities, and the effects on MAPK/AP-1 signaling and associated pathways.

This study is the very first report on molecular effects of monanchocidin B (**2**), monanchocidin C (**3**), ptilomycalin A (**4**), monanchomycalin B (**5**), normonanchocidin D (**6**), urupocidin A (**7**), and pulchranin A (**8**) in cancer cells.

## 2. Results and Discussion

### 2.1. Inhibition of EGF-Induced Malignant Transformation of Murine Epithelial Cells

First, the ability of the alkaloids **1**–**4**, **7**, and **8** to prevent the EGF-induced neoplastic transformation and colony formation was studied using a model of murine epithelial JB6 P^+^ Cl41 cells and standard anchorage independent malignant transformation assay [34,39,40]. JB6 cells can be sensitive (JB6 P^+^) or not sensitive (JB6 P^−^) to tumor promoters, such as EGF or 12-*O*-tetradecanoylphorbol-13-acetate (TPA), leading to malignant transformation of the cells and resulting in anchorage-independent colony formation upon stimulation with these agents (Figure 1B) [40,41]. Anchorage-independent growth of cancer cells in vitro has been characterized as a hallmark of the tumor phenotype, particularly with respect to metastatic potential (reviewed in [42]). Remarkably, marine substances **1**–**4**, **7**, and **8** were able to inhibit EGF-induced malignant transformation and anchorage-independent colony formation of JB6 P^+^ Cl41 cells at low micro- and nanomolar concentrations (Figure 1B,C). Interestingly, in these experiments, active concentrations (INCC_50_) were three- to eight-fold lower than the corresponding cytotoxic concentrations (Table 1). In fact, the highest IC_50_/INCC_50_ ratios were observed for compounds **2** and **7** (Figure 1C). This high ratio of IC_50_/INCC_50_ suggests potent cancer-preventive activity of the compound without induction of side effects caused by its cytotoxic properties, although this awaits further confirmation in vivo.

**Figure 2:**
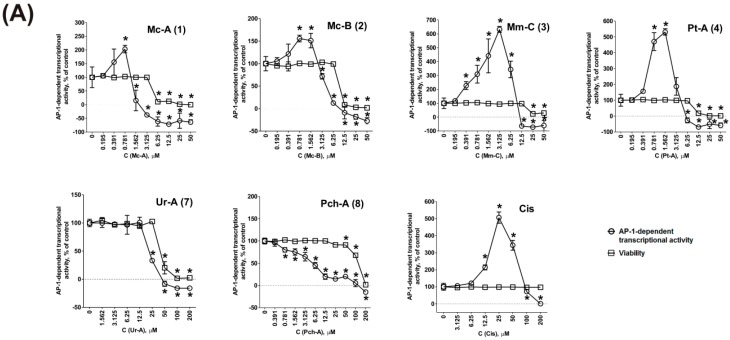

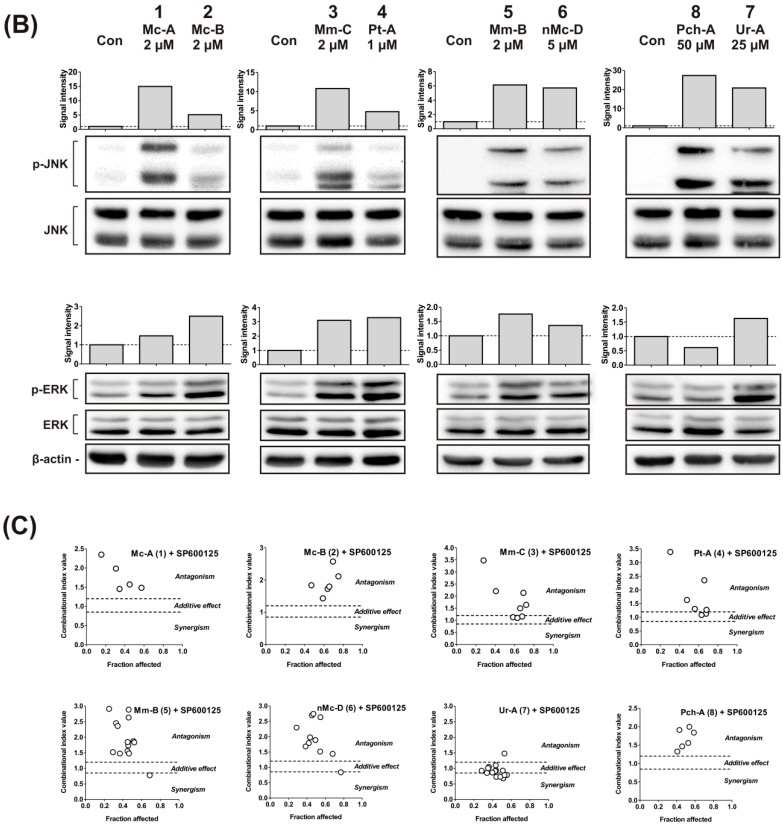
Effect of alkaloids **1**–**8** on MAPK/AP-1 signaling. (**A**) Effect on basal AP-1-dependent transcriptional activity (**○**) and viability (□) of JB6 Cl41 cells stably expressing a luciferase reporter gene controlled by the AP-1 DNA binding sequence after 12 h of treatment. Cell viability was measured using MTT assay; (**B**) effect on the activation of JNK1/2 and ERK1/2. JB6 P^+^ Cl41 cells were treated with the compounds **1**–**8** at the indicated concentrations for 48 h, and the level of protein expression was assessed by Western blotting. The intensities of p-JNK1/2 and p-ERK1/2 signals were quantified with Quantity One 4.6 software (Bio-Rad, Hercules, CA, USA) and normalized against the signals of total JNK1/2 and ERK1/2, correspondently; and (**C**) the effect of SP600125 (specific JNK1/2 inhibitor) on the survival of JB6 P^+^ Cl41 cells treated with compounds **1**–**8**. Cells were co-treated with different concentrations of the individual drugs or their combination for 48 h. Cell viability was measured by MTT assay and the combinational index (CI) values were calculated with CompuSyn software (v.1.0., ComboSyn Inc., Paramus, NJ, USA) using the Chou-Talalay method. The ratio of the substances and the effects are presented in the Supplementary Materials. * indicates a statistically significant difference (*p* < 0.05) from the control value.

**Figure 3:**
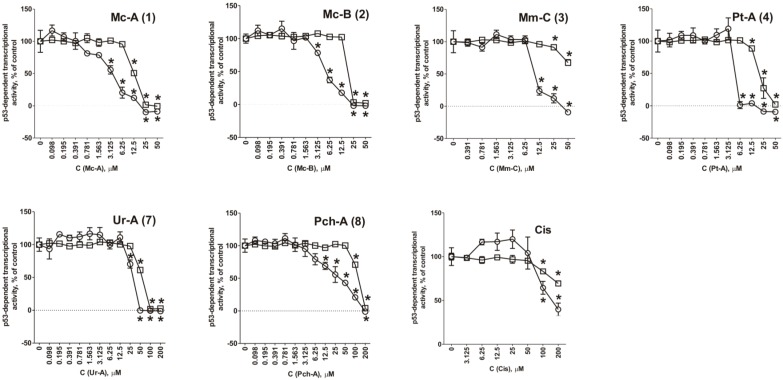
Effect alkaloids **1**–**8** on basal p53-dependent transcriptional activity (**○**) and viability (□) of JB6 Cl41 cells stably expressing a luciferase reporter gene controlled by the p53 DNA binding sequence after 12 h of treatment. Cell viability was measured using MTT assay. * indicates a statistically significant difference (*p* < 0.05) from the control value.

Note, due to the small amount available, substances **5** and **6** were used only in a limited number of experiments. Thus, these compounds were not examined in the current experiments (inhibition of EGF-induced malignant transformation, Figure 1B,C), as well as in experiments on AP-1- and p53-dependent transcriptional activities modulations (Figure 2A and Figure 3, correspondently).

### 2.2. Effect on MAPK/AP-1 Signaling

Malignant transformation of JB6 P^+^ Cl41 cells involves the activation of the nuclear factor activator protein-1 (AP-1) [43,44,45], while blocking AP-1-transcriptional activity, can mediate inhibition of EGF-induced cell transformation [44,46,47]. Mitogen-activated protein kinases (MAPK) JNK1/2 and ERK1/2 are well-known upstream players of the AP-1 activation pathway. JNK1/2 and ERK1/2 promote the AP-1 transcriptional factor through the activation of expression and stabilization of Fos and Jun [48,49,50]. We, therefore, investigated the effects of the sponge alkaloids on MAPK/AP-1 signaling using the well-established model of JB6 Cl41 cells stably expressing a luciferase reporter gene controlled by an AP-1 binding sequence [35,36,51].

First, we evaluated the effect of the alkaloids **1**–**4**, **7**, and **8** on AP-1-dependent transcriptional activity (Figure 2A). Indeed, pulchranin A (**8**) potently suppressed AP-1 transcriptional activity with a significant inhibition at non-cytotoxic doses of at least 70-fold lower than the IC_50_ of the compound (Table 1). Interestingly, in cells treated with urupocidin A (**7**), AP-1-dependent transcriptional activity was suppressed concurrently with the reduction of cell viability (Figure 2A). Therefore, we conclude that urupocidin A (**7**) does not affect AP-1-transcriptional activity in the living cells. Surprisingly, ptilomycalin A-like compounds **1**–**4** induced AP-1 transcriptional activity at pre-cytotoxic concentrations (after 12 h of treatment) in stably-transfected JB6 Luc AP-1 cells. Consequently, AP-1 suppression is not involved in the inhibition of EGF-induced malignant transformation by compounds **1**–**4** and **7** (although it might be involved in the effect of pulchranin A (**8**)).

In addition, the transcription factor AP-1, apart from malignant transformation, is involved in regulation of a wide range of cellular processes (for review see [48,50,52]). Among others, AP-1 is known to be involved in the induction of programmed death of cancer cells [48,50,53]. It was reported that some cancer preventive and cytotoxic natural compounds, including those of terrestrial biological sources (e.g., the anticancer drug vinblastine [54,55], the cancer preventive flavonoids kaempferol and genistein [56], and the anti-inflammatory drug tolfenamic acid [57]) and those of marine origin (e.g., the alkaloids 3- and 10-bromofascaplysins [58], the 3-demethylubiquinone Q2, and its synthetic analogs [33,59], and the cancer preventive terpenoid dactylone [34]), as well as some DNA-damaging agents [60], can activate AP-1. Thus, AP-1 activation in our experiments may be involved in the cytotoxic effects of these drugs (at least for compounds **1**–**4**) (Figure 2A). In line with this, the effects of compounds **1**–**4** were similar to the effect observed upon treatment with cisplatin, which also induced a dose-dependent increment of AP-1 activity at pre-cytotoxic concentrations (Figure 2A). In contrast, alkaloids **7** and **8**, which did not activate AP-1 were significantly less cytotoxic (Table 1), underlining the pro-apoptotic role of AP-1 activation by guanidine alkaloids.

Next, we evaluated the effects of the compounds on upstream mitogen-activated protein kinases (MAPK) JNK1/2 and ERK1/2 in JB6 P^+^ Cl41 (Figure 2B). We have shown that all compounds, except pulchranin A (**8**), at concentrations in the range of the respective IC_50_s induce activation (phosphorylation) of JNK1/2 and ERK1/2. Pulchranin A (**8**) did not lead to ERK1/2 activation (Figure 2B). Therefore, we postulate that the kinases JNK1/2 and ERK1/2 are involved in the cellular responses to the drugs **1**–**8** (with the exception of ERK1/2 for pulchranin A (**8**)).

To prove the importance of JNK1/2 activation for the cytotoxic effect of the guanidine alkaloids **1**–**8** we performed combinational assays with the well-established JNK1/2 inhibitor SP600125 (Figure 2C). At the active concentrations, SP600125 itself was cytotoxic for JB6 P^+^ Cl41 cells. Therefore, the ability of SP600125 to inhibit (antagonize) the cytotoxic effects of the investigated alkaloids was analyzed using the Chou-Talalay method [61]. We could show that SP600125 is able to antagonize the cytotoxic effect of compounds **1**–**6** and **8**, but not of urupocidin A (**7**) (Figure 2C), although JNK1/2 activation was observed for this compound as well (Figure 2B). This finding strongly suggests that JNK1/2 is important for the cytotoxic effect of ptilomycalin A-like guanidine alkaloids (**1**–**6**), as well as pulchranin A (**8**), but not of urupocidin A (**7**).

### 2.3. Role of p53 in Cytotoxic Effect of the Guanidine Alkaloids

It is known that about 50% of tumors harbor mutant p53 with abrogated tumor-suppressive function [62]. Thus, compounds which are able to induce apoptosis independently of p53 activity could be useful, especially in the treatment of tumors that have lost their p53 function [62]. To investigate the role of p53 in cytotoxicity of compounds **1**–**4**, **7**, and **8** we used a well-established JB6-Luc p53 (PG-13) cell model [35,36,51]. These cells stably express a luciferase reporter gene controlled by the p53 DNA binding sequence. We could not observe any activation of p53-dependent transcriptional activity upon the treatment with any of the compounds **1**–**4**, **7**, and **8** (Figure 3). Therefore, we conclude that cell death induced by these alkaloids occurs independently of transcriptional activity of the tumor suppressor protein p53. The observed effects were similar to those induced by cisplatin, which is known to be able to induce p53-independent apoptosis in normal human fibroblasts, as well as in cancer cells [63,64,65,66]. Before, crambescidin-816 from the marine sponge *Crambe crambe*, being structurally similar to ptilomycalin A, was reported to increase the expression of p53 pathway components [28]. However, our current finding suggests that active p53 is not required for cytotoxicity of the guanidine alkaloids **1**–**4**, **7**, and **8** and, therefore, we postulate that these compounds may be effective in cells and tumors bearing mutant p53.

### 2.4. Induction of Programmed Cell Death and Cell Cycle Arrest in Cancer Cells

**Figure 4:**
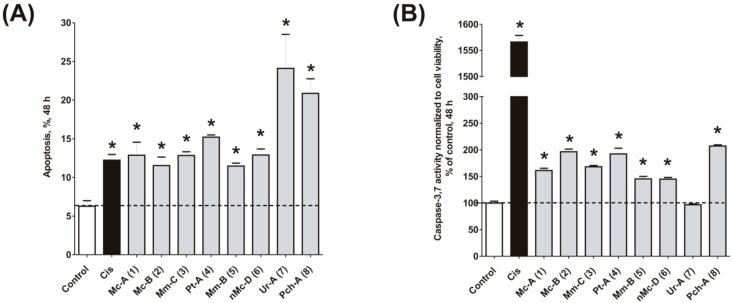
Pro-apoptotic activity of alkaloids **1**–**8** in human cancer cells. (**A**) Analysis of DNA fragmentation in HeLa cells treated with compounds **1**–**8** for 48 h at the concentrations of IC_50_. The number of cells with fragmented DNA was assessed with flow cytometry and assumed as the sub-G1 population in cycle analysis; and (**B**) analysis of caspase-3/7 activity in HeLa cells under the treatment with the compounds **1**–**8** at the concentrations of IC_50_ for 48 h. Cisplatin was used as a positive control. Concentrations used corresponded to IC_50_ for HeLa cells as presented in Table 1. * indicates a statistically significant difference (*p* < 0.05) from the control value.

To confirm the relevance of cytotoxic properties of the compounds **1**–**8** we examined their effects on cell viability (Table 1) and cell cycle progression (Table 2) in human cancer HeLa cells. Ptilomycalin A-like alkaloids **1**–**6** exhibited comparable cytotoxicities, however, monanchocidin B (**2**) appeared to be the most active compound in human cancer cells (Table 1). All compounds induced DNA fragmentation in malignant cells, indicating induction of programmed cell death (Figure 4A). Although most of the drugs induced activation of caspase-3/7 in HeLa cells at their respective IC_50_s, these effects were distinctly less pronounced when compared to treatment with the conventional chemotherapeutic agent cisplatin (Figure 4B). These findings support our previous data on induction of non-apoptotic cells death by monanchocidin A (**1**) in GCT cells [14] and suggest that the structurally-related alkaloids **2**–**6**, as well as the structurally-distinct compounds urupocidin A (**7**) and pulchranin A (**8**) may have similar non-apoptotic mechanisms of action. Interestingly, HeLa cells treated with urupocidin A (**7**) did not exhibit caspase-3/7 activation (Figure 4B), although the percentage of cells containing fragmentated DNA was quite high (Figure 4A). This may suggest that caspase-related apoptotic pathways are not involved in the cellular response to urupocidin A (**7**) at all while, in the case of monanchocidin A (**1**), we found it to be of minor importance [14].

Interestingly, normonanchocidin D (**6**), which—in contrast to the compounds **1**–**5**—does not have a spermidine or oxyspermidine motif in its structure, still holds comparable cytotoxic activity (Table 1, Figure 3 and Figure 4). In addition, the cytotoxic activity of compounds containing spermidine (**3**–**5**) or oxyspermidine (**1**, **2**) did not differ significantly from each other. Consequently, we assume that this motif is most likely not important/essential for the cytotoxicity of ptilomycalin A-like compounds. It has been previously suggested before that the presence of spermidine in the molecule of ptilomycalin A-like compounds leads to the inhibition of eIF5A hypusination and, consequently, contributes to cell death or growth suppression [15]. Our present research, therefore, suggests that this speculation was not correct.

Additionally, we investigated the effects of the marine compounds on the cell cycle progression of human cervical carcinoma cells (Table 2). All the ptilomycalin A-like compounds **1**–**6** induced S-phase arrests, while treatment with urupocidin A (**7**), having a different chemical structure, induced G2/M-arrest (Table 2). Pulchranin A (**8**) did not alter cell cycle phase distribution of HeLa cells (Table 2). These data are in line with the previous reported S-phase cell cycle arrest induced by ptilomycalin A-like crambescidin-800 [23], as well as the effects of monanchocidin A on different human cancer cell line [14].

## 3. Materials and Methods

### 3.1. Reagents and Antibodies

The guanidine alkaloids: monanchocidin A (**1**) and monanchocidin B (**2**) [10], monanchomycalin C (**3**) and ptilomycalin A (**4**) [17], monanchomycalin B (**5**) [9], normonanchocidin D (**6**) [22], urupocidin A (**7**) [21], and pulchranin A (**8**) [11] were isolated from marine sponge *Monanchora pulchra* as described earlier. The sponge was collected in the Sea of Okhotsk (Pacific Ocean, Russian Far East). The precise coordinates of the sponge collections have been published before (see above). The purity of each compound was proved by ^1^H NMR (nuclear magnetic resonance), MS (mass spectrometry), HPLC (high performance liquid chromatography), and TLC (thin-layer chromatography) data, as well as by measuring of optical rotations. Anisomycin and cisplatin (*cis*-diamminedichloroplatinum (II), 1 mg/mL) were purchased from NeoCorp (Weilheim, Germany), SP600125. Primary and secondary antibodies used were: anti-β-Actin-HRP (goat pAb, sc-1616, Santa Cruz, 1:10,000), anti-ERK (mouse mAb, #9107, Cell Signaling Technology, 1:2000), anti-JNK (rabbit mAb, #9258, Cell Signaling Technology, 1:1000), anti-phospho-ERK (rabbit mAb, #4377, Cell Signaling Technology, 1:1000) anti-phospho-JNK (rabbit mAb, #4668 Cell Signaling Technology, 1:1000), secondary anti-mouse IgG-HRP (sheep, NXA931 GE Healthcare, 1:10,000), secondary anti-rabbit IgG-HRP (goat, #7074, Cell Signaling Technology, 1:5000).

### 3.2. Cell Culture

The human cancer cell line HeLa (cervical carcinoma) was obtained from ATCC (Manassas, VA, USA). The JB6 P^+^ Cl41 mouse epidermal cell line and its stable transfectants, JB6-Luc AP-1 and JB6-Luc p53 (PG-13), cells were kindly provided by Prof. Zigang Dong, Hormel Institute, University of Minnesota, MN, USA. Cells were cultured in monolayers at 37 °C and 5% CO_2_ in l-glutamine-supplied RPMI (for HeLa cells) or MEM (for JB6 P^+^ Cl41, JB6-Luc AP-1, and JB6-Luc p53 cells) media, containing 10% FBS (fetal bovine serum) and 1% penicillin/streptomycin (Invitrogen, Paisley, UK). Cells were continuously kept in culture for a maximum of three months, and were routinely inspected microscopically for stable phenotype and regularly checked for contamination with mycoplasma.

### 3.3. In Vitro MTT-Based Drug Sensitivity Assay

Cytotoxicity of the individual compounds was evaluated by MTT assay. Experiments were performed as previously described [67]. In brief, 6000 cells/well were seeded in a 96-well plate, incubated overnight, and treated with the drugs in 100 µL/well of fresh media. After 48 h of treatment cell viability was measured spectrophotometrically using MTT (3-(4,5-dimethylthiazol-2-yl)-2,5-diphenyltetrazolium bromide) reagent.

### 3.4. Anchorage-Independent Neoplastic Transformation (Colony Growth Assay)

The cancer preventive activity of alkaloids **1**–**8** was evaluated using an anchorage-independent neoplastic transformation assay. The experiment was performed as described before [35]. In brief, EGF (10 ng/mL) was used to induce neoplastic transformation of JB6 P^+^ Cl41 cells. The assay was carried out in six-well tissue culture plates. Mouse JB6 P^+^ Cl41 cells (8 × 10^3^ cells/mL) were treated with various concentrations of the compounds in 1 mL of 0.33% basal medium Eagle (BME)-agar containing 10% FBS over 3 mL of 0.5% BME-agar containing 10% FBS and various concentrations of the tested drugs. The plates were incubated at 37 °C in 5% CO_2_ atmosphere for 14 days, following the scoring of cell colonies using an Olympus CKX31 inverted research microscope (Olympus, Center Valley, PA, USA).

### 3.5. Determination of the Effect of Сompounds on the Basal Transcriptional Activity of AP-1 and p53

The effect of the marine guanidine alkaloinds on the basal transcriptional activities of AP-1 or p53 was evaluated using JB6 Cl41 cell lines stably expressing a luciferase reporter gene controlled by an AP-1- or p53-DNA binding sequence [35,36,51], as described previously, with slight modifications [36]. Briefly, cells were pre-incubated overnight in 96-well plates (20 × 10^3^ cells/well) in culture medium (100 μL/well). Then, the medium was replaced with fresh medium containing different concentrations of compounds. After incubation for 12 h, cells were lysed for 1 h at RT with lysis buffer (0.1 M PBS (pH 7.8), 1% Triton X-100, 1 mM DTT, 2 mM EDTA). Then, 30 μL of lysate from each well was transferred into a plate for luminescent analysis, and luciferase activity was measured using luciferase assay d-luciferin-based buffer (100 μL/well) and the Luminoscan Ascent Type 392 microplate reader (Labsystems, Helsinki, Finland).

### 3.6. Cell Cycle and DNA Fragmentation Analysis

The cell cycle distribution was analyzed by flow cytometry using PI staining as described before [68]. In brief, cells were pre-incubated overnight in six-well plates (0.2 × 10^6^ cells/well) and treated with the investigated drugs. After 48 h of treatment, cells were trypsinized, fixed with 70% EtOH/H_2_O (*v*/*v*) at −20 °C overnight, stained with buffer containing propidium iodide (PI) and RNase, and analyzed with a BD Bioscience FACS Calibur analyzer (BD Bioscience, Bedford, MA, USA). The data were analyzed using BD Bioscience Cell Quest Pro v.5.2.1. software (BD Bioscience). Cells containing fragmented DNA were detected as a sub-G1 population.

### 3.7. Examination of Antagonistic Effects of JNK1/2 Inhibitor SP600125 on the Cytotoxicity of the Tested Compounds

Determination of synergistic, antagonistic, or additive effects of SP600125 on the activity of investigated compounds were analyzed using the Chou-Talalay method [61]. The experiment was performed as described before with slight modifications [14,38]. Drugs were combined in a non-constant molar ratio and the data were generated using MTT assay. Cells were pre-treated for with 20 µM or 40 µM of SP600125 in the 50 µL/well culture media for 1 h. Then, drug-containing or drug-free media was added to wells (50 μL/well, up to a total volume 100 μL/well). Cells were incubated for 48 h and the cell viability was measured with MTT assay as described above. The combinational index (CI) was calculated with the CompuSyn v.1.0 software (ComboSyn, Inc., Paramus, NJ, USA). Synergism is defined as a CI < 0.85, whereas antagonism is defined by a CI > 1.2. A CI value of 0.85–1.2 reflects an additive effect. The amount/dosages of the compounds used for combination treatment are indicated in Supplementary Materials. All experiments were performed in triplicate.

### 3.8. Western Blotting

Preparation of protein extracts for Western blotting was performed as described previously [35]. In brief, for Western blotting, 1 × 10^6^ cells/well were seeded in Petri dishes (ø 6 cm TC Dish (Sarstedt, Numbrecht, Germany), 5 mL/dish), incubated overnight, and treated with drugs for 48 h in 2 mL/dish. Cells were harvested using a cell scraper, washed, and lysed with the Western blotting lysis buffer [68]. Lysates were frozen overnight at −20 °C and then centrifuged. Protein concentration in the supernatants was determined by Bradford assay [69].

Western blotting was performed as described before [67]. Total protein extracts (20–30 µg/sample) were subjected to electrophoresis in SDS-polyacrylamide gels at 120 V, and transferred from gel to a 0.2 µm pore PVDF membrane. The membrane was blocked and incubated with the primary antibody according to the manufacturers’ protocol. After washing, the membranes were incubated with the corresponding secondary antibody for 1 h at RT. Signals were detected using the ECL chemiluminescence system (Thermo Scientific, Rockford, IL, USA) according to the manufacturer’s protocol. Relative optical density of the signal intensity of the bands was quantified with Quantity One 4.6 software (Bio-Rad, Hercules, CA, USA). The signals of p-JNK/1/2 and p-ERK1/2 were normalized against the signals of total JNK/1/2 and ERK1/2, correspondingly.

### 3.9. Caspase-3/7 Activity Assay

The enzymatic activity of caspase-3 and -7 were measured using Caspase-Glo^®^ 3/7 Assay Kit (Promega, Madison, WI, USA). Six thousand cells per well were seeded in a 96-well sterile, white, flat-bottom plate in 100 µL/well and incubated overnight. Then the media was changed with 90 µL/well of fresh media containing investigated substances at various concentrations. After 48 h of treatment enzymatic caspase-3/7 activity or cells viability was measured. To measure caspase-3/7 activity, 90 µL/well of Caspase-Glo^®^ 3/7 reagent was added and the plates were incubated for 1 h at RT in the dark. The luminescence was measured using an Infinite F200PRO reader (TECAN, Männedorf, Switzerland). Cells viability was measured using MTT assay (see Table 1) with slight modifications: cells were incubated with MTT reagent for 2 h, then the media was carefully aspirated, plates were dried for 1 h at RT, and 90 µL/well of DMSO were added to dissolve the formazan crystals. Then 90 µL of formazan/DMSO solution were transferred into new transparent plate and optical density was read with a photometer. The enzymatic activity of caspase-3 and -7 were normalized to the cell viability at the correspondent concentration of the drug.

### 3.10. Statistical Analysis

Statistical analyses were performed using GraphPad Prism software v. 5.01 (GraphPad Prism software Inc., La Jolla, CA, USA). Data are presented as mean ± SEM (standard error of the mean). All of the experiments were performed in triplicates and repeated at least three times. The unpaired Student’s *t*-test was used for comparison of two groups. Differences were considered to be statistically significant and marked with an asterisk (*) if *p* < 0.05.

## 4. Conclusions

**Figure 5:**
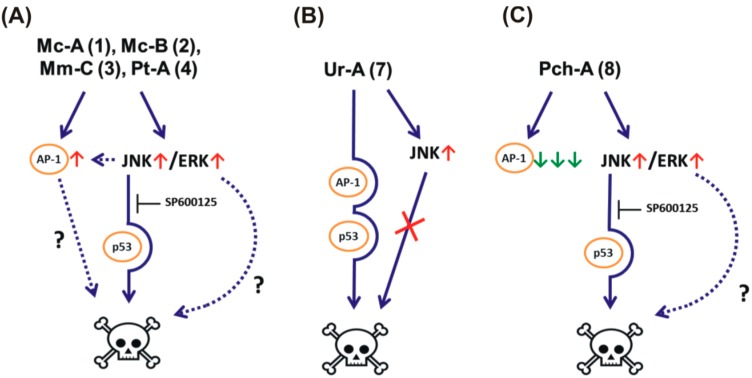
Suggested modes of action of the compounds **1**–**4** (**A**); **7** (**B**); and **8** (**C**).

The observed effects are summarized in Table 3 and Figure 5A–C. Ptilomycalin A-like compounds **1**–**4** activated JNK1/2 and ERK1/2, following AP-1-activation and caused p53-independent programmed cell death and S-phase cell cycle arrest (Figure 5A). Structurally-distinct urupocidin A (**7**) induced JNK1/2 and ERK1/2 phosphorylation, as well as p53-independent programmed cell death and G2/M-phase cell cycle arrest, however, JNK1/2 activation did not contribute to the cytotoxicity of the alkaloid. Additionally, p53-independent cell death induced by urupocidin A (**7**) was not accompanied by alteration of AP-1 transcriptional activity, as well as by caspase-3/7 activation. This suggests a distinctly different mechanism of cytotoxic action of urupocidin A (Figure 5B). Another structurally-distinct alkaloid pulchranin A (**8**) induced JNK1/2 activation leading to p53-independent programmed cell death without cell cycle arrest induction. However, pulchranin A (**8**) did not activate ERK1/2 and was able to strongly suppress AP-1-transcriptional activity at non-cytotoxic concentrations (Figure 5C). An important finding was the ability of compounds **1**–**4**, **7**, and **8** to inhibit the EGF-induced neoplastic transformation of JB6 P^+^ Cl41 cells. In summary, our results suggest that guanidine alkaloids from marine sponge *Monanchora pulchra* hold potential to eliminate human cancer cells, as well as to prevent cancer cell formation and spreading, which awaits further in vivo confirmation. This research provides the very first insight in the mechanisms of action of compounds **2**–**8** and relative alkaloids in cancer cells.

## Figures and Tables

**Table 1:** IC_50_ of alkaloids **1**–**8** in human cancer HeLa cells and mouse non-malignant JB6 P^+^ Cl41 cell lines after 48 h of treatment.

No.	Compound	Cytotoxic Activity, IC_50_ [µМ], 48 h	
		HeLa Cells	JB6 P^+^ Cl41 Cells
**1**	Monanchocidin A (Mc-A)	1.39	2.01
**2**	Monanchocidin B (Mc-B)	0.58	1.36
**3**	Monanchomycalin C (Mm-C)	1.84	1.31
**4**	Ptilomycalin A (Pt-A)	1.1	0.5
**5**	Monanchomycalin B (Mm-B)	1.5	1.72
**6**	Normonanchocidin D (nMc-D)	2.1	5.2
**7**	Urupocidin A (Ur-A)	28.7	27
**8**	Pulchranin A (Pch-A)	51	58
	Cisplatin	4.75	30.2

**Table 2:** Cell cycle analysis of human cancer HeLa cells treated with alkaloids **1**–**8** at the concentrations of IC_50_ for 48 h.

-	Control	1 (Mc-A)	2 (Mc-B)	3 (Mm-C)	4 (Pt-A)	5 (Mm-B)	6 (nMc-D)	7 (Ur-A)	8 (Pch-A)	Cis
**G1-phase**	69.5 ± 1	62 ± 0.7	63.8 ± 1.5	65.6 ± 1.8	65.4 ± 0.4	66.3 ± 0.9	71.4 ± 0.1	65.3 ± 3	73 ± 1.5	7 ± 0.6
**S-phase**	13.8 ± 0.4	17 ± 0.7 *	21 ± 0.1 *	21.5 ± 1.4 *	22.6 ± 2 *	23.7 ± 1.2 *	17.6 ± 0.2 *	10.6 ± 0.8	11.2 ± 1.5	15.6 ± 3.5
**G2/M-phase**	16.3 ± 0.7	21.4 ± 1.3 *	14.9 ± 0.8	12.1 ± 2.1	12.2 ± 1.5	10.3 ± 1.1	10.5 ± 0.8	23.7 ± 0.5 *	15.7 ± 0.8	77.6 ± 3.7 *
**Cell cycle arrest**		S, G2/M	S	S	S	S	S	G2/M	no	G2/M
**Raw data**	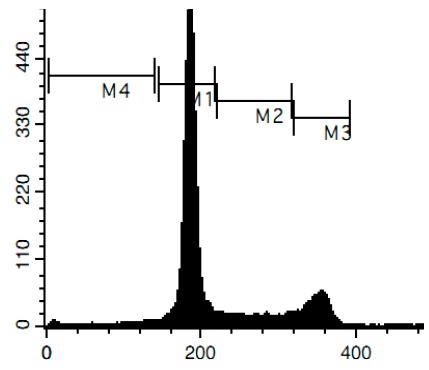	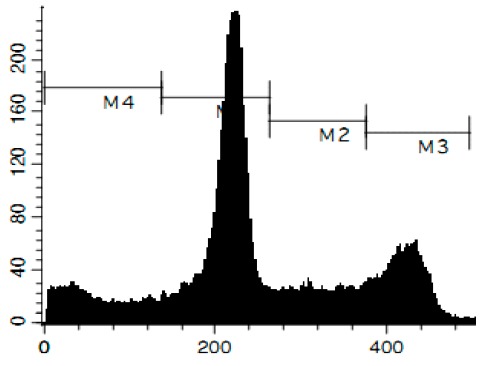	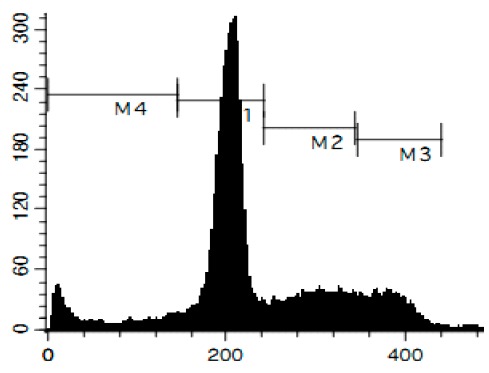	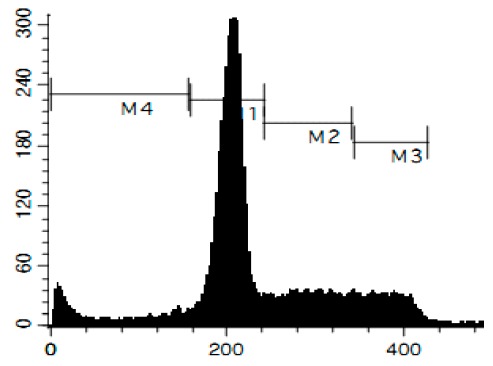	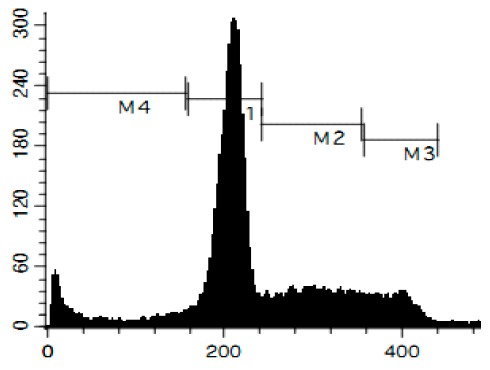	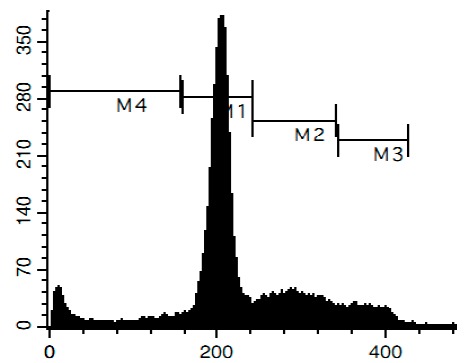	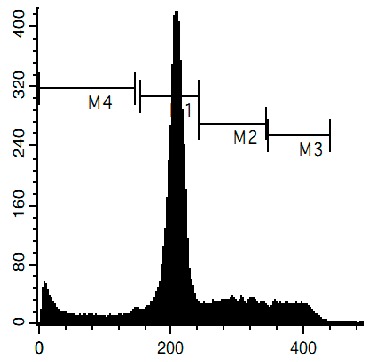	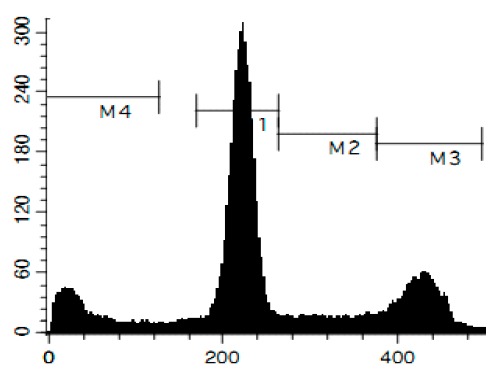	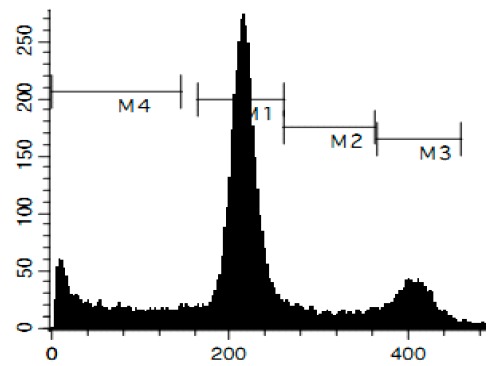	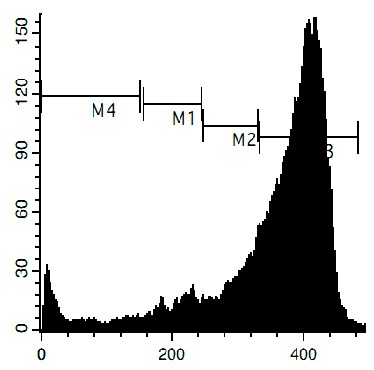

* indicates values significantly different from control.

**Table 3:** Summary of observed effects of compounds **1**–**8**.

Effect	1 (Mc-A)	2 (Mc-B)	3 (Mm-C)	4 (Pt-A)	5 (Mm-B)	6 (nMc-D)	7 (Ur-A)	8 (Pch-A)	Cis
Effect on DNA fragmentation at IC_50_	↑↑	↑↑	↑↑	↑↑	↑↑	↑↑	↑↑↑	↑↑↑	↑↑
Effect on caspase-3/7 activity at IC_50_	↑	↑	↑	↑	↑	↑	no	↑↑	↑↑↑
Cell cycle arrest	S, G2/M	S	S	S	S	S	G2/M	no	G2/M
Effect on AP-1-transcriptional activity	↑	↑	↑	↑	-	-	no	↓	↑
Effect on ERK phosphorylation	↑	↑	↑	↑	↑	↑	↑	no	-
Effect on JNK phosphorylation	↑	↑	↑	↑	↑	↑	↑	↑	-
Effect of SP600125 (JNK inhibitor) on the drug cytotoxic activity	↓	↓	↓	↓	↓	↓	no	↓	-
Activation of p53-transcriptional activity	no	no	no	no	no	no	no	no	no

“↑”—activation up to two-fold in comparison with control; “↑↑”—activation up to three-fold in comparison with control; “↑↑↑”—activation >three-fold in comparison with control; “↓”—inhibition; “no”—no effect; “-”—the drug was not tested in this assay.

## Supplementary Materials

The following are available online at www.mdpi.com/1660-3397/14/7/133/s1, the ratio of the substances **1**–**8** and JNK1/2 inhibitor SP600125 as well as the effects.
