# Supplementary material for: Guanidine Alkaloids from the Marine Sponge Monanchora pulchra Show Cytotoxic Properties and Prevent EGF-Induced Neoplastic Transformation in Vitro
Source: Mar Drugs. 2016 Jul 15;14(7):133. doi: 10.3390/md14070133 (PMC4962023; doi:10.3390/md14070133)
Supplement: Supplementary file 1 [file marinedrugs-14-00133-s001.pdf]

Supplementary Material: Guanidine Alkaloids from the Marine Sponge *Monanchora pulchra* Show Cytotoxic Properties and Prevent EGF-Induced Neoplastic Transformation in Vitro

Sergey A. Dyshlovoy, Kseniya M. Tabakmakher, Jessica Hauschild, Regina K. Shchekaleva, Katharina Otte, Alla G. Guzii, Tatyana N. Makarieva, Ekaterina K. Kudryashova, Sergey N. Fedorov, Larisa K. Shubina, Carsten Bokemeyer, Friedemann Honecker, Valentin A. Stonik and Gunhild von Amsberg

The effect of SP600125 (specific JNK1/2 inhibitor) on the survival of JB6 P^+ Cl41 cells treated with compounds **1**–**8**. Cells were co-treated with indicated concentrations of the individual drugs or their combination for 48 h. Cell viability was measured by the MTT assay and the combinational index (CI) values were calculated with CompuSyn software using Chou-Talalay method. Mc-A, monanchocidin A (**1**); Mc-B, monanchocidin B (**2**), Mm-C, monanchomycalin C (**3**); Pt-A, ptilomycalin A (**4**); Mm-B, monanchomycalin B (**5**); nMc-D, normonanchocidin D (**6**); Ur-A, urupocidin A (**7**); Pch-A, pulchranin A (**8**). The effect of the treatments vary from 0 (all cells are alive) to 1 (all cells are dead).

Dose Mc-A, μM	Dose SP600125, μM	Effect	CI
0.25	20.0	0.155	2.34884
0.5	20.0	0.345	1.45284
1.0	20.0	0.573	1.48387
0.25	40.0	0.307	1.98479
0.5	40.0	0.45	1.57143

Dose Mc-B, μM	Dose SP600125, μM	Effect	CI
0.75	20.0	0.47	1.83752
1.5	20.0	0.638	1.72573
3.0	20.0	0.693	2.57508
0.75	40.0	0.589	1.4313
1.5	40.0	0.656	1.79942
3.0	40.0	0.749	2.11484

Dose Mm-C, μM	Dose SP600125, μM	Effect	CI
0.75	20.0	0.28	3.4779
1.5	20.0	0.583	1.13072
3.0	20.0	0.678	1.15795
6.0	20.0	0.719	1.64088
0.75	40.0	0.407	2.20717
1.5	40.0	0.629	1.10073
3.0	40.0	0.658	1.49944
6.0	40.0	0.691	2.1381

Dose Pt-A, μM	Dose SP600125, μM	Effect	CI
0.5	20.0	0.304	3.3885
1.0	20.0	0.559	1.30904
2.0	20.0	0.676	1.13367
4.0	20.0	0.658	2.35328
0.5	40.0	0.477	1.63314
1.0	40.0	0.63	1.08641
2.0	40.0	0.681	1.27275

Dose Mm-B, μM	Dose SP600125, μM	Effect	CI
0.375	20.0	0.293	1.52167
0.75	20.0	0.367	1.47834
1.5	20.0	0.448	1.55816
3.0	20.0	0.461	2.63426
0.375	40.0	0.251	2.91645
0.75	40.0	0.326	2.45096
1.5	40.0	0.457	1.73874
3.0	40.0	0.461	2.88906
0.75	20.0	0.343	2.36287
1.5	20.0	0.464	1.48077
3.0	20.0	0.512	1.87456
6.0	20.0	0.687	0.77791
0.75	40.0	0.293	4.5777
1.5	40.0	0.45	1.84516
3.0	40.0	0.521	1.83676

Dose nMc-D, μM	Dose SP600125, μM	Effect	CI
0.625	20.0	0.287	2.29674
1.25	20.0	0.42	1.79187
2.5	20.0	0.454	2.69936
5.0	20.0	0.678	1.43814
0.625	40.0	0.384	1.67978
1.25	40.0	0.433	1.97571
2.5	40.0	0.469	2.74596
5.0	40.0	0.767	0.83689
1.25	20.0	0.492	1.88748
2.5	40.0	0.493	3.74941
1.25	40.0	0.543	1.51716
2.5	40.0	0.545	2.63287

**Dose Ur-A, μM**	**Dose SP600125, μM**	**Effect**	**CI**
3.125	20.0	0.283	0.92335
6.25	20.0	0.339	0.8535
12.5	20.0	0.397	0.86526
25.0	20.0	0.434	1.09976
3.125	40.0	0.352	1.00969
6.25	40.0	0.441	0.7273
12.5	40.0	0.483	0.72863
25.0	40.0	0.528	0.782
6.25	20.0	0.36	1.0469
12.5	20.0	0.435	0.93691
25.0	20.0	0.509	0.92798
50.0	20.0	0.527	1.47783
6.25	40.0	0.427	0.91371
12.5	40.0	0.515	0.6809
25.0	40.0	0.554	0.78736

**Dose Pch-A, μM**	**Dose SP600125, μM**	**Effect**	**CI**
12.5	20.0	0.406	1.32739
25.0	20.0	0.429	1.9124
50.0	20.0	0.537	1.99839
12.5	40.0	0.459	1.46527
25.0	40.0	0.524	1.55852
50.0	40.0	0.586	1.83779
